# Diffuse Alveolar Hemorrhage Associated with Epithelioid Angiosarcoma of the Lung: A Challenging Diagnosis

**DOI:** 10.1155/2023/5553209

**Published:** 2023-06-16

**Authors:** Felix Wuest, Andreas Gebhardt, Claudia Grosswendt, Sergej Griff, Antonina Zhilina, Samantha Taber, Jens Kollmeier, Torsten T. Bauer

**Affiliations:** ^1^Department of Pneumology, Lungenklinik Heckeshorn, Helios Klinikum Emil von Behring, Walterhoeferstrasse 11 14165 Berlin, Germany; ^2^Institute of Diagnostic and Interventional Radiology, Nuclear Medicine and Molecular Imaging, Helios Klinikum Emil von Behring, Berlin, Germany; ^3^Institute of Pathology, Helios Klinikum Emil von Behring, Berlin, Germany; ^4^Department of Thoracic Surgery, Lungenklinik Heckeshorn, Helios Klinikum Emil von Behring, Berlin, Germany

## Abstract

A 68-year-old patient presented with persistent hemoptysis and weight loss. A CT scan showing diffuse bilateral ground-glass opacities and nodules was followed by bronchoscopy. While diffuse alveolar hemorrhage (DAH) could be seen, specimens obtained during bronchoscopy did not provide conclusive histological findings. The decision was made to conduct video-assisted wedge resection, after which histological examinations revealed the diagnosis of bifocal nodular manifestation of an epithelioid angiosarcoma in the lung. Being a rare entity even among sarcomas, these kinds of tumors can be primary lung tissue angiosarcomas or metastatic lesions with primaries in places like the skin, breast, and heart. Treatment usually includes chemotherapy, but prognosis remains grim. This case highlights that in DAH, uncommon causes should be considered, and sufficient probe gathering is the key to early diagnosis and treatment.

## 1. Introduction

Malignancy is a rare cause of diffuse alveolar hemorrhage (DAH) [1]. Among malignant tumors, sarcomas represent a rather uncommon entity, with angiosarcomas accounting for around 1% of these soft tissue sarcomas. Furthermore, primary epithelioid angiosarcomas of the lung are far less prevalent than metastatic lesions with primaries in places like the skin, breast, heart, liver, and gastrointestinal tract [2]. We report a patient with DAH caused by epithelioid angiosarcoma of the lung.

## 2. Case Presentation

**Figure 1:**
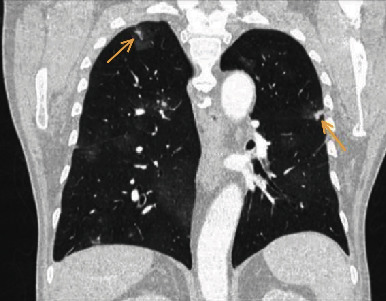
Frontal lung window. Multiple bilateral ground-glass attenuation mostly centered around small nodules (arrowheads).

**Figure 2:**
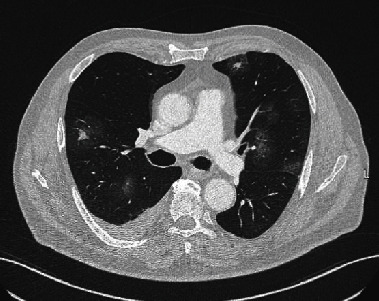
Axial lung window. The ground-glass opacities likely represent peritumoral alveolar hemorrhage, caused by the fragility of neovascular tissue. Pleural effusion is on the right.

We present a 68-year-old patient who was referred to our hospital with severe weight loss and persistent hemoptysis for almost three months. The patient had a smoking history of 30 pack years but no other known exposure to toxic agents. Preexisting conditions included peripheral artery disease, hypertension, and chronic back pain. An abnormal chest X-ray led to a diagnostic bronchoscopy in another hospital, but the cause of bleeding could not be determined (Figure 1). After admission to our hospital, we ordered a CT scan of the lung, which showed multiple, bilateral medium sized nodules, surrounded by areas of ground-glass opacities (Figure 2). Some of the lesions appeared to infiltrate the pleural tissue, and a right-sided pleural effusion was present. Laboratory tests showed mild normocytic anemia (13.0 g/l), elevated WBC (13.9 G/l), and increased CRP (55 mg/dl), while levels of ANCAs, ACPA, and ANA were negative. Although a differential diagnosis of Goodpasture syndrome was initially considered, normal anti-GBM levels made this cause of DAH less likely. Thoracocentesis revealed a hemorrhagic pleural effusion with no evidence of malignant cells. Another diagnostic bronchoscopy demonstrated diffuse alveolar hemorrhage, while a BAL taken from the 8th segment on the right showed a low percentage of macrophage-laden hemosiderin. A biopsy obtained from the left lower lobe revealed an unspecific inflammation containing eosinophils and lymphocytes. As hemoptysis continued, prednisolone therapy was initiated, but even after several days of treatment, no improvement was seen. As there was still no conclusional histological finding, the decision was made to secure larger biopsies via video-assisted thoracoscopic wedge resection. Guided by radiological suspect area, probes were removed from segments 1/3/9/10 of the right lung.

**Figure 3:**
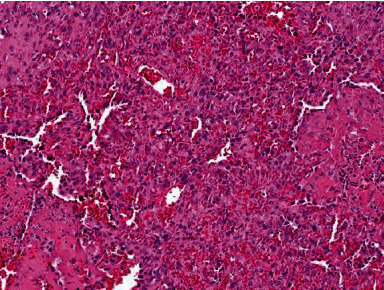
Sheet-like proliferation and variably formed vascular channels with erythrocytes, lined by high-grade atypical cells.

**Figure 4:**
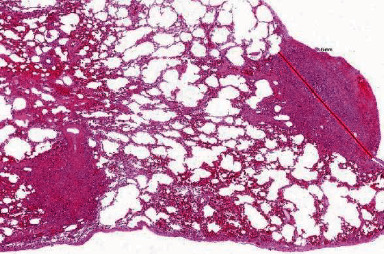
Bifocal nodular proliferates of epithelioid angiosarcoma.

**Figure 5:**
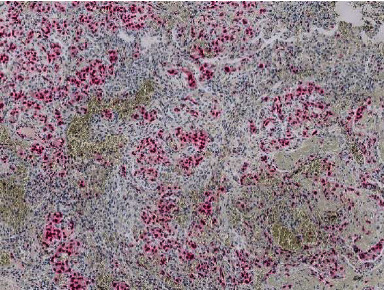
Nuclear staining for transcriptional regulator ERG.

Histological examination revealed small airways, regular lung parenchyma with an alveolar basic structure, and bronchioles lined by regular respiratory epithelium. Microscopic examination also demonstrated subpleural and intraparenchymal nodular proliferates of anastomosing vessels and solid sheets with hemorrhage and patchy necrosis, with maximum diameters of 5 mm (Figure 3). The neoplasms contained high-grade atypical epithelioid cells with slightly unrounded nuclei and small nucleoli, as well as inconspicuous mitotic figures and abundant cytoplasm (Figure 4). Immunostainings revealed strong positivity for CD31 and ERG (Figure 5) and negativity for pan cytokeratin (MNF116). The proliferative index determined by Ki67% (Mib-1) was ca. 20%. Based on these findings, the diagnosis of a bifocal nodular manifestation of an epithelioid angiosarcoma of the lung parenchyma was established. The patient was then transferred to the nearest sarcoma center. Because of persisting hemoptysis and dropping red blood cell counts, chemotherapy was administered before completing a full staging. A CT scan of thorax/abdomen after the second cycle of gemcitabine and docetaxel showed partial remission of the lung tumor mass while no other tumor lesions were detected.

## 3. Discussion

Epithelioid angiosarcoma is a rare subspecies of sarcoma, accounting for around 1% of all sarcomas. The vast majority of angiosarcomas appearing in the lung are actually metastatic lesions from other locations such as the skin, heart, liver, bone, or gastrointestinal tract [2]. Primary angiosarcomas of the lung are extremely rare; a 2015 review on the topic found only 32 cases that had been reported in literature until then [3].

Due to the very low incidence of this disease, it is difficult to establish risk factors; these may, however, include Lucite plombage, chronic empyema, and tuberculous pyothorax [4]. Males with a median age of 55.9 years seem to be affected more frequently. The most common symptoms include hemoptysis or hemoptoe, followed by cough, chest pain, and weight loss [3]. Diffuse alveolar hemorrhage, which is defined by hemoptysis, anemia, and radiological diffuse alveolar infiltrates, usually is not caused by malignant tumors but instead by Goodpasture syndrome, polyangiitis with granulomatosis, systemic lupus erythematosus, or coagulation disorders [1]. In our patient, these causes were all ruled out by laboratory testing. Our case revealing DAH secondary to epithelioid angiosarcoma in the lung aligns with other case reports of pulmonary hemorrhage as the main manifestation [5, 6]. A 2001 study by Adem et al. concluded that angiosarcoma should be included in the differential diagnosis of diffuse pulmonary hemorrhage, especially in young adults [7].

As demonstrated in this case, abnormal findings in chest radiography are insufficient for establishing a diagnosis, but they often serve as an impulse to initiate further examinations. Radiological features in CT scans usually include nodules (in metastatic lesions, commonly subpleural), often surrounded by ground-glass opacities (halo sign), diffuse infiltrations, pleural effusions, and sometimes hemothorax. Large specimens are usually necessary to provide conclusive pathological findings so that in most cases, the initial bronchoscopy is followed by surgical resection [3]. It is important to emphasize surgical tissue sampling as the method of choice in establishing the diagnosis of pulmonary angiosarcoma. Definitive diagnosis is based on immunohistochemical and histopathological findings. Characteristics include infiltrative lesions composed of large oval or round cells with eosinophilic nucleoli. Hemorrhage, mitosis, and necrosis are often observed as well. Immunohistochemically, the neoplastic cells may show reactivity for endothelial cell markers such as CD31, CD34, factor VIII, epithelial markers, and vimentin [8].

Likely, due to the low incidence, data on effective treatment remains scarce, and a standard of treatment does not exist. Based on what we know so far, surgical resection is likely the best treatment; however, due to the aggressiveness of these tumors, most patients present in advanced stages. Regarding chemotherapy, a regimen of docetaxel and gemcitabine has been reported to result in complete radiographic response [9]. Recent literature also suggests that checkpoint inhibitors (e.g., pembrolizumab) may have a positive impact on long-term survival in angiosarcomas of different locations, including the lung [10, 11]. The reported effectiveness of recombinant interleukin-2 remains unclear. Although there is some indication that it may provide a benefit, it was investigated in combination with radiotherapy, which angiosarcomas seem to be sensitive to [12, 13]. Unfortunately prognosis continues to be grim with median overall survival rates of around 11 months [14].

## 4. Summary

We herein report a case of DAH, secondary to an epithelioid angiosarcoma of the lung. If common causes for DAH are ruled out and bronchoscopy also fails to provide conclusive pathological material, diagnostic surgical resection can help to ensure a timely diagnosis and initiation of treatment.

## Data Availability

All relevant data have been included in the manuscript. Further details and information about the case are available upon request.
